# The effect of sung speech on socio-communicative responsiveness in children with autism spectrum disorders

**DOI:** 10.3389/fnhum.2015.00555

**Published:** 2015-10-29

**Authors:** Arkoprovo Paul, Megha Sharda, Soumini Menon, Iti Arora, Nayantara Kansal, Kavita Arora, Nandini C. Singh

**Affiliations:** ^1^National Brain Research CentreGurgaon, India; ^2^International Laboratory of Brain, Music and Sound Research (BRAMS), University of MontrealMontreal, QC, Canada; ^3^Children First Mental Health InstituteNew Delhi, India

**Keywords:** autism, socio-communicative responsiveness, song, joint attention, eye contact

## Abstract

There is emerging evidence to demonstrate the efficacy of music-based interventions for improving social functioning in children with Autism Spectrum Disorders (ASD). While this evidence lends some support in favor of using song over spoken directives in facilitating engagement and receptive intervention in ASD, there has been little research that has investigated the efficacy of such stimuli on socio-communicative responsiveness measures. Here, we present preliminary results from a pilot study which tested whether sung instruction, as compared to spoken directives, could elicit greater number of socio-communicative behaviors in young children with ASD. Using an adapted single-subject design, three children between the ages of 3 and 4 years, participated in a programme consisting of 18 sessions, of which 9 were delivered with spoken directives and 9 with sung. Sessions were counterbalanced and randomized for three play activities—block matching, picture matching and clay play. All sessions were video-recorded for *post-hoc* observational coding of three behavioral metrics which included performance, frequency of social gesture and eye contact. Analysis of the videos by two independent raters indicated increased socio-communicative responsiveness in terms of frequency of social gesture as well as eye contact during sung compared to spoken conditions, across all participants. Our findings suggest that sung directives may play a useful role in engaging children with ASD and also serve as an effective interventional medium to enhance socio-communicative responsiveness.

## Introduction

Impairments in the socio-communicative domain are a hallmark feature of Autism Spectrum Disorders (ASD) (Kanner, 1943a; Zwaigenbaum et al., 2005; American Psychiatric Association, 2013). These impairments are reflected in behaviors such as the inability to orient socially, understanding and use of social gestures, gaze following, eye contact, imitation as well as the capacity to initiate and/or respond to joint attention. An extensive body of research has established these early emerging social behaviors as important building blocks for a typical developmental trajectory (Mundy et al., 1990; Charman et al., 2003). More specifically, these behaviors are critical in initiating and maintaining social relationships and verbal language development. A number of studies have shown that there are significant challenges in the development of skills associated with these socio-communicative behaviors in children with autism (Mundy et al., 1986; Charman et al., 1997; Dawson et al., 1998, 2004; Lozier et al., 2014). Consequently, such behaviors are important targets for early intervention in children with ASD (Warreyn et al., 2005; Jones et al., 2006; Leekam and Ramsden, 2006; Whalen et al., 2006).

An emerging practice for targeting socio-communicative impairments in ASD is the use of music- and song-based interventions (Lim, 2010; Wan et al., 2011; Simpson et al., 2013). Historically, the use of music has always been associated with increased engagement and a preserved domain of functioning. In the earliest scientific account, Kanner had noted the exceptional musical capacity of children with autism (Kanner, 1943a,b). Subsequent investigations further confirmed that children with autism showed a preference for musical stimuli (Thaut, 1988; Buday, 1995). These were also accompanied by numerous anecdotal reports that described the unique and profound effect music has on children with autism (Sacks, 2007). Other studies of musical abilities have demonstrated enhanced skills such as perfect pitch and good melodic memory in children with ASD (Heaton et al., 2008; Molnar-Szakacs and Heaton, 2012; Ouimet et al., 2012). In the domain of affect and music, Heaton et al. (1998) showed that children with autism had a good understanding of the affective implications of musical mode and were able to pair happy and sad faces with excerpts of music in major and minor keys, suggesting that the inability to identify emotions in social stimuli like faces, did not apply to the musical domain. It is also important to note that musical preferences in individuals with autism develop early in life (Allen et al., 2009) and responsiveness to music is found to remain preserved in adults on the autism spectrum, though it is often underestimated due to their reduced ability to articulate it (Allen et al., 2013). However, integrated reviews of the literature on music therapy (MT) interventions have consistently noted music's potential to support the social and affective development of young children with autism (Whipple, 2004; Kaplan and Steele, 2005; Gold et al., 2006; Accordino et al., 2007; Simpson and Keen, 2011).

Recently, studies from the neuroimaging domain have also provided compelling biological evidence showing preserved neural activity for music processing in children with ASD (Lai et al., 2012; Sharda et al., 2015). For instance, a neuroimaging study by Caria et al. (2011), showed that individuals with ASD recruit regions involved in emotion and reward processing while listening to happy and sad musical excerpts, similar to neurotypical controls. On the other hand, two studies (Lai et al., 2012; Sharda et al., 2015) showed that brain regions that show decreased activation during speech stimulation in ASD vs. controls showed greater activation during song stimulation. In fact, the study by Sharda et al. (2015), also demonstrated that fronto-temporal connectivity in the brain remains intact during perception of sung but not spoken words in children with ASD (Sharda et al., 2015). These findings provide robust neurobiological support for the use of music and song stimuli for therapeutic purposes and suggest that the sung stimulus might be a powerful medium to engage a child with ASD.

Since ASD presents a unique condition where socio-communicative impairments and enhanced music perceptual abilities coexist, clinicians have often attempted to capitalize on the musical strengths of individuals to compensate for their social difficulties (Alvin, 1978; Alvin and Warwick, 1992; Vaiouli et al., 2015). Recently, MT has been classified as an emerging evidence-based practice, useful in teaching individual skills or goals, through the use of specific musical components, such as songs, rhythm, and movement (Geretsegger et al., 2014; Thaut et al., 2015). Although MT has long been used for rehabilitation of neurological disorders (Wan et al., 2010b) and cognitive development (Paul et al., 2012), its potential and validation to improve social, cognitive and motor skills for individuals with autism (American Music Therapy Association, 1999, 2003; Kaplan and Steele, 2005; Molnar-Szakacs and Heaton, 2012) is still an emerging field. The literature pertaining to the use of music as an interventional medium in ASD has focused predominantly on socio-communicative behaviors, with music being consistently used to explore the development of social skills in children (Duffy and Fuller, 2000; Finnigan and Starr, 2010). More recently, a novel music intervention based on auditory-motor mapping has been developed to aid expressive language development for non-verbal children with ASD (Wan et al., 2011). Another study comparing infant-directed speech with infant-directed song on the levels of engagement and learning outcomes (Simpson et al., 2015), used spoken and sung conditions embedded in a computer-based communication intervention, developed to teach receptive labeling in children with autism. Combined together, the above studies provide both behavioral and neurobiological motivation for use of music, especially song, as an effective tool for improving socio-communicative responsiveness in individuals with autism (Gold et al., 2006; Simpson et al., 2013; Geretsegger et al., 2014).

Based on this premise, the aim of the current study was to further investigate the effects of singing on socio-communicative responsiveness in children with ASD. More, specifically, efficacy of sung-directives to improve eye contact and social responsiveness in children with ASD were studied and the potential of intoned vocalizations and singing as an interventional medium, suited for the clinic and easily adaptable for home and classroom settings, was examined. In contrast to previous cross-over or group level designs, we employed an adapted single subject research design to control for within subject variability (Barlow and Hayes, 1979; Barlow and Herson, 1984; Scruggs et al., 1987; Horner et al., 2005; Kennedy, 2005). The main goal was to assess the efficacy of song as a medium of intervention in ASD, given its intrinsic motivational value. We hypothesized that sung instructions may act as a communicative scaffold for children with ASD and consequently be more engaging and elicit greater number of socially responsive behaviors in participants, as compared to spoken directives.

## Methods

### Participants

Three children, all boys (mean age = 3.36 years, *SD* = 0.21) participated in this study. All three children were diagnosed using the Diagnostic and Statistical Manual of Mental Disorders-5 (DSM 5, American Psychiatric Association, 2013) and International Classification of Diseases-10 (ICD 10, World Health Organization, 1992) criteria by experienced medical professionals. Standard assessment measures including the Social Responsiveness Scale (SRS 2, Constantino and Gruber, 2012), Childhood Autism Rating Scale (CARS II, Schopler et al., 1980), parental reports and direct child observations were used to confirm the diagnoses. The Vineland Adaptive Behavior Scale (VABS II, Sparrow et al., 2005) was administered to assess adaptive behavior and socio-communicative skills. Detailed demographics are provided in Table 1. To be eligible for participation in the study, the participants had to be (1) formally diagnosed with ASD by a practicing physician, (2) chronologically aged between 3 and 5 years, (3) able to participate in the 18 sessions of the research programme, and (4) without any other comorbid neurological or psychiatric diagnosis. The participants were selected based on parental consultation and informed consent procedures approved by the Institutional Ethics Committee. Detailed information for each child is provided below.

**Table 1:** **Behavioral profile and standardized test scores for all participants**.

	Child A	Child B	Child C
**PARTICIPANT DETAILS:**			
Age (years)	3.33	3.58	3.17
Gender	Male	Male	Male
**ASSESSMENTS:**			
**Childhood Autism Rating Scale (CARS) II**			
T- score	41	52	53
Severity group	Mild to moderate	Severe	Severe
**Social Responsiveness Scale (SRS) II**			
Total T-score	75	89	79
Severity group	Moderate	Severe	Severe
Social awareness T score	74	83	60
Social cognition T score	73	82	77
Social communication T score	74	89	77
Social motivation T score	62	76	66
Restricted interests and repetitive behavior T score	82	94	96
DSM-5 compatible scores			
Social communication and interaction T score	73	87	74
Restricted interests and repetitive behavior T score	82	94	96
**Vineland Adaptive Behavior Scale (VABS) II**			
Adaptive behavior composite score	72	71	67
Adaptive level	Moderately low	Moderately low	Low
Communication domain score	76	54	59
Daily living skills domain score	75	81	77
Socialization domain score	68	77	66
Motor skills domain score	82	88	81

*Summary of behavioral profile of the participants*.

#### Child A

Child A was 3 years 4 months old when the study commenced. He had a repertoire of few words, such as “hello,” names of objects and people which he could use in 2–3 word sentence combinations for social greetings and need-based communication. He was a socially-oriented child with evident joint attention in high to moderate interest activities. He also displayed delayed echolalia and template language, and was quick to adapt to repetitive routines and patterns in play and social interaction. Child A was the higher functioning child amongst the three participants with a CARS score of 41 (mild to moderate) and SRS of 75. He often showed neutral affect and occasionally produced echolalic words or phrases.

#### Child B

Child B was 3 years 7 months old at the start of the programme. He had extremely low functional language skills and frequently uttered vocalizations without any apparent communicative intent. Child B had a CARS score of 52 (severe). He displayed low joint attention in social interactions and often avoided eye contact. He used to engage primarily in solitary play. He had sensory processing difficulties and emotional dysregulation, which often manifested in disruptive behaviors. His awareness of self and others was low, and sitting tolerance and ability to attend to table top activities was also difficult.

#### Child C

Child C was 3 years 2 months old when the study started. He was averbal at the beginning of the study and showed minimal to none signs of communicative intent via verbal or vocal modalities. Child C was severely affected by autism with a CARS score of 53. He usually maintained a neutral disposition and avoided eye contact. He was reported to be a child who tended to remain in a world of his own and did not show interest in any social activity. His responses could occasionally be elicited in a therapeutic setting by high interest sensory routines.

### Procedure

The study used an adapted single subject research design of AB type (Barlow and Hayes, 1979; Barlow and Herson, 1984; Scruggs et al., 1987; Horner et al., 2005; Kennedy, 2005). In a single subject design participants serve as their own controls; and thus it was preferred for this study to account for the within-subject variability across the two conditions—(A) spoken directives (considered the baseline condition) and (B) sung directives (considered the treatment condition), as individuals with ASD largely vary on their behavioral profiles. This was a “proof of concept” study to test our hypothesis that song may be more efficient than spoken directives to act as a communicative scaffold and enhance socio-communicative responsiveness in young children with ASD.

#### Programme

The programme consisted of 18 sessions over a period of 3 months for each child. Each session consisted of a (A) spoken or (B) sung condition. Three activities were used for all sessions. Each activity was used in both sung and spoken conditions. Each session was of 3–4 min duration per condition. Similar directives such as “Hello,” “Look at me,” “Let's match pictures,” “Let's play with blocks” etc. were used in all sessions. Both spoken and sung sessions contained similar semantic content and only differed in the intonation of the directives. This was to ensure that any differences in behavior could be attributed to the musical nature of the directives used. Representative spectrograms for sample stimuli [refer to Supplementary Audio Clips S1, S2 for (A) spoken and (B) sung conditions] are attached in Supplementary Figure S1 to further illustrate this. Every child took part in 9 sessions with spoken directives and 9 sessions with sung directives, counterbalanced and randomized for the three play activities such as block matching, picture matching and clay play. All conditions and activities were further randomized to account for day-to-day variability in each child's performance.

The activities were chosen as the preferred play activities as reported by the therapists. The materials used for these play activities consisted of colored wooden blocks of different shapes, picture matching board games and synthetic modeling clay. Each session took place in a secluded room at the intervention clinic with the participant and the trained therapist seated across from each other at a table. A second caregiver videotaped the sessions from an adjacent position to the participant using a video camera and played no role in conducting the sessions. The therapist delivered the spoken and sung directives during the spoken and sung sessions, respectively while engaging the child in play activities. All sessions were video-recorded for *post-hoc* observational coding of three behavioral metrics including performance, frequency of social gesture, and eye contact as described below.

#### Independent variables

The study examined the participant's socio-communicative responsiveness within two experimental conditions: (A) the baseline spoken directive condition and (B) the treatment sung directive condition. In both conditions each participant was presented with bids within a play context by the therapist with the goal of having the child respond in a socially appropriate manner. The participant-therapist interaction took place in the following format: (1) the therapist would greet and/or present a preferred play material and initiate a communicative bid; (2) the participant was expected to respond; and (3) the participant's response, if correct/appropriate, would be reinforced by applause. The only difference between (A) the baseline spoken and (B) the treatment sung condition was the intonation of directives used by the therapist as illustrated in the Supplementary Figure S1; while all other elements of the session such as the semantic content, session structure, and settings were unaltered.

#### Dependent variables

Several dependent variables were operationally defined in order to characterize the participants' response to the experimenter's communicative bids. (1) “Performance” on each session was measured as a percentage of correct responses with respect to the total number of instructional directives presented to the child during that session. This measure was used as a non-social measure of responsiveness to assess the participants' overall performance and comprehension abilities associated with each play session. Socio-communicative responsiveness was measured using two distinct behaviors–social gesture and eye contact. (2) “Social gesture” was defined as the child's physical response to social greeting such as “hi five” and was measured as a percentage of instances of such social touch with respect to the total number of opportunities received from the experimenter. (3) “Eye contact” was measured as the percentage of frequency of eye contact made by the child with respect to the total number of occurrences of name calling by the experimenter. All these measures were evaluated using videos for each session by a trained rater using a custom-made rating scheme (Hooker, 2013).

#### Reliability

An independent second rater trained in behavioral coding but blind to the purpose of the study rated 30% of the video recordings. These videotapes were randomly selected and the three different behavioral measures defined above were coded from each video. Cohen's Kappa value was calculated for all three behavioral measures to assess the inter-rater reliability. There was substantial agreement on looking behavior (kappa = 0.69) and social gesture (kappa = 0.70) whereas the kappa value on performance (kappa = 0.82) represented almost perfect agreement (Viera and Garrett, 2005). Only the behavioral measures recorded by the primary rater were used for data analysis.

### Results

**Figure 1:**
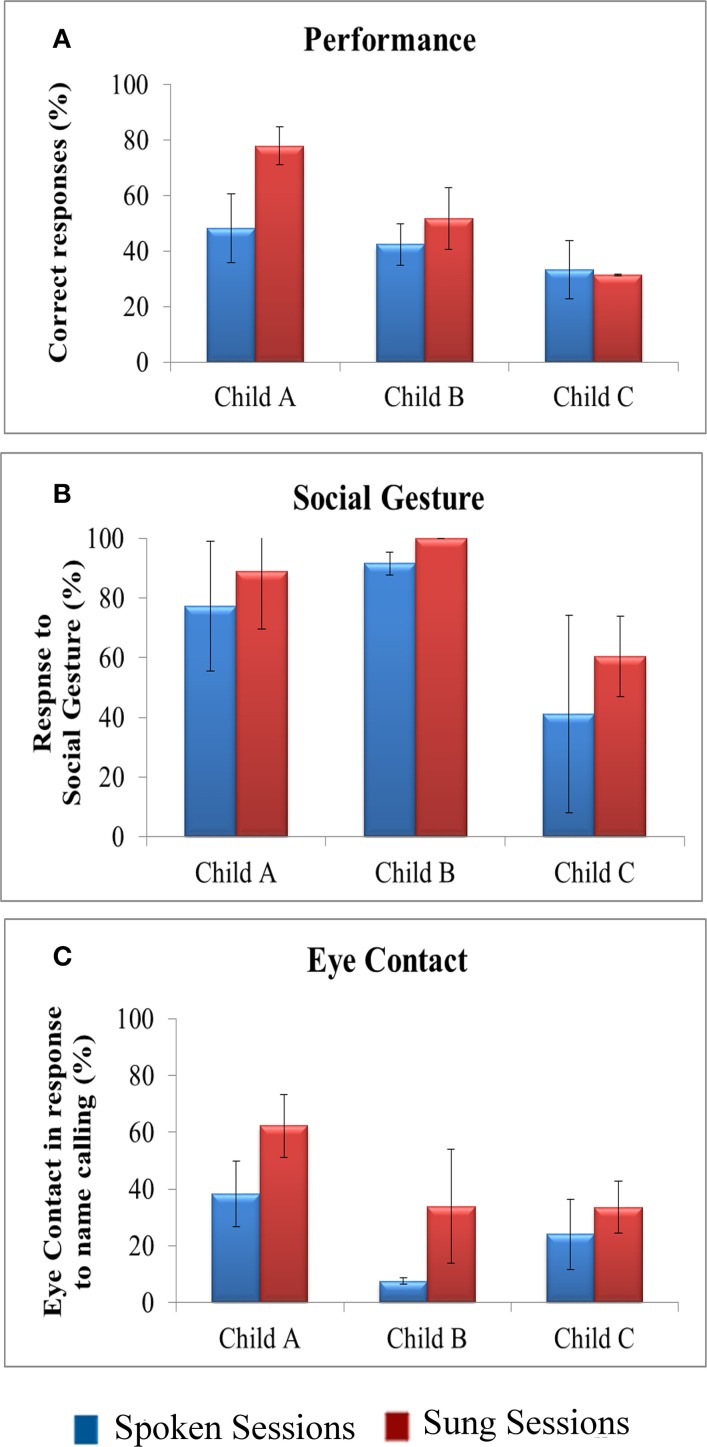
**Comparison of behavioral measures in spoken vs. (B) sung sessions**. The figure shows the comparison of overall percent scores on the behavioral metrics **(A)** performance, **(B)** social gesture, and **(C)** eye contact for each participant in spoken (blue) vs. sung (red) conditions across all 18 sessions. The means of all three behavioral measures across the sessions revealed an overall increase in sung sessions compared to baseline spoken conditions.

The results of measured behaviors for all three participants in (A) baseline spoken vs. (B) treatment sung conditions are shown in Figure 1. All participants scored higher in the treatment (sung) condition compared to the baseline (spoken) on all measures including performance, social gesture and eye contact. Child A performed much better in sung condition with a mean of ~78% correct responses to instructional directives when compared to 48% mean correct responses for spoken sessions. Child B was a little lower on accuracy with a mean of ~52% in the spoken condition and a mean of 42% in the sung condition. The performance of Child C was quite low and comparable across both spoken and sung conditions (with a mean of 33% in spoken and a mean of 31% in sung condition).

The data also indicate that there was a trend of enhanced responsiveness to social gestures in the sung condition, as compared to baseline spoken condition, for all three participants. Child A responded to social gestures with a mean of 77% in spoken and ~89% in sung conditions. Child B showed ceiling effects with very high level of responses, particularly in this behavioral category, both in spoken (a mean of 91%) and sung (a mean of 100%) conditions. Finally, Child C also showed a similar pattern with lower responses in (mean of 41%) spoken conditions compared to sung (mean of 60%) conditions, although the variability for performance on spoken conditions (*SD* = 33%) was very high.

A similar trend of increased frequency of eye contact in response to name calling across the sung sessions was observed. Child A made an average of 38% eye contact in the spoken sessions compared to a mean of 62% in sung sessions. Child B responded with a mean of 7.5% eye contact in spoken condition as compared to a mean of ~34% in sung condition, though with a high variability (*SD* = 20). Child C also showed an increase in eye contact from a mean of 24% in spoken condition to a mean of 33% for sung condition. Overall, the observational analysis of the videos indicated increased socio-communicative responsiveness in terms of both frequency of social gesture as well as eye contact during the sung as compared to the spoken condition, across all 3 participants.

**Figure 2:**
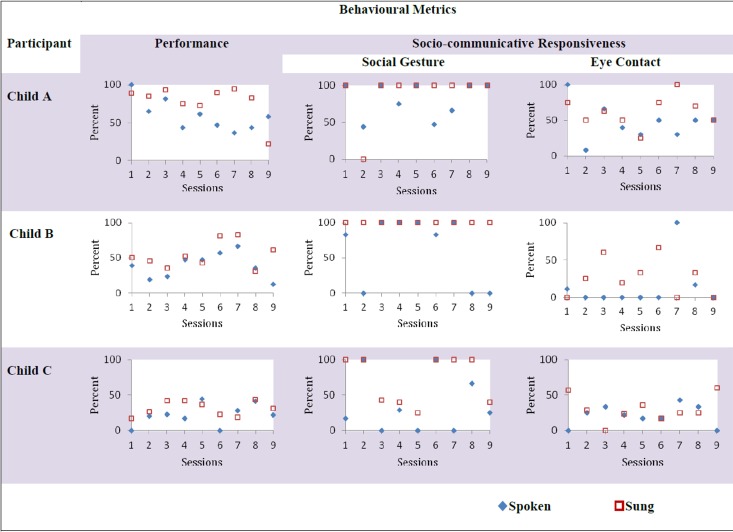
**Trajectory of behavioral measures (performance, social gesture, and eye contact) compared for the three participants (Child A, Child B, Child C) in spoken and sung sessions**. The top panel represents three behavioral metrics of performance (percentage of correct responses), social gesture (percentage of frequency of social gestures made in response to social bids such as “hi five”) and eye contact (percentage of frequency of eye contact made in response to name calling) across 9 sung (red) and 9 spoken (blue) sessions for all 3 activities, randomized, and counterbalanced across 18 sessions for child A. The lower panels show the same for child B and child C, respectively. For all participants, the scores for all measures in the sung sessions were greater than (or equal to) the spoken sessions- performance (Child A-7 out of 9 sessions, Child B-7 out of 9 sessions, Child C-7 out of 9 sessions), social gesture (Child A-8 out of 9, Child B-all sessions, Child C- all sessions), and eye contact (Child A-6 out of 9 sessions, Child B-6 out of 9 sessions, Child C-6 out of 9 sessions).

Nevertheless, there was a high degree of variability in the data, revealed by the trajectory of performance for all the participants across 18 sessions, comprising of 9 spoken sessions and 9 sung sessions (Figure 2). Visual inspection was used to examine changes in measured behavior as it is considered to be the most appropriate and most commonly used method of analysis in single-subject design research (Horner et al., 2005; Kennedy, 2005). For all participants, the scores for all measures in the sung sessions were greater than (or equal to) the spoken sessions- performance (Child A-7 out of 9 sessions, Child B-7 out of 9 sessions, Child C-7 out of 9 sessions), social gesture (Child A-8 out of 9, Child B-all sessions, Child C- all sessions), and eye contact (Child A-6 out of 9 sessions, Child B-6 out of 9 sessions, Child C-6 out of 9 sessions). Moreover, the means of all three behavioral measures across the sessions revealed an overall increase in sung sessions compared to baseline spoken conditions (Figure 1).

**Figure 3:**
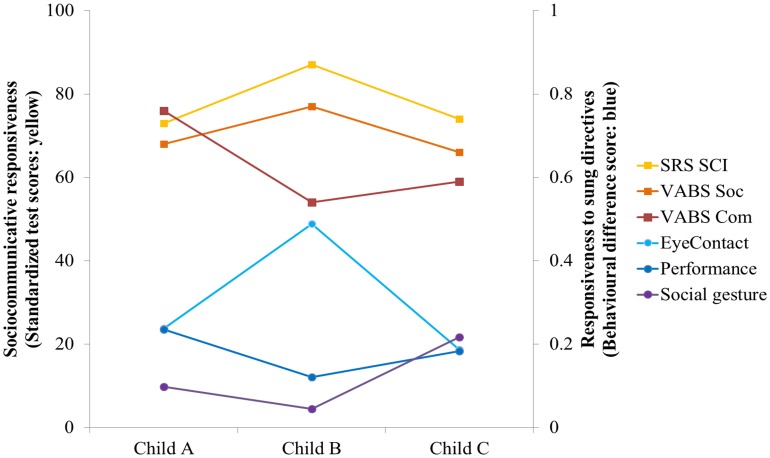
**Comparison of responsiveness to sung directives as a function of socio-communicative skills for all participants**. The responsiveness to sung directives is defined as the “difference score” [(sung − spoken)/(sung + spoken)]. The difference scores for performance and socio-communicative responsiveness such as social gesture and eye contact (shown in blue) are plotted against socio-communicative skills or standardized test scores such as VABS and SRS (shown in yellow) for all three participants. Child B with higher standardized test score in VABS socialization and SRS SCI domains showed an increased responsiveness to sung directives as reflected by the difference score for socio-communicative responsiveness in comparison with the other two participants. Child C who had a comparatively lower standardized test scores in VABS socialization and communication and SRS SCI domains also showed comparable responsiveness to sung directives for social gesture, eye contact and performance. VABS, Vineland Adaptive Behavior Scale (subscales—Soc, socialization; Com, communication). SRS, Social Responsiveness Scale (subscales—SCI, social communication and interaction T score; RRB, Restricted Interests and Repetitive Behavior T score).

To further characterize the profile of participants to assess responsiveness to sung vs. spoken directives, VABS socialization and communication domain scores and SRS social communication and interaction (SCI) scores for each child were compared with their overall “responsiveness to sung words” for performance, social gesture and eye contact measures (Figure 3). This measure of responsiveness was calculated as a difference score: [(sung – spoken)/(sung + spoken)] for all three measures. Child B, with the higher standardized test score in VABS socialization and SRS SCI domains, showed an increased responsiveness to sung directives as reflected by the difference score for socio-communicative responsiveness in comparison with the other two participants. Interestingly, Child C who had a comparatively lower standardized test scores in VABS socialization and communication and SRS SCI domains also showed comparable responsiveness to sung directives for social gesture, eye contact, and performance.

### Discussion

Findings from the current study indicate the effectiveness of using singing and song-based directives in improving socio-communicative responsiveness of young children with ASD. Such song-based directives can be implemented as a medium of communication in interventional programmes at home, clinics as well as school-based settings to facilitate communication and interactions between individuals with ASD and their parents and care givers to help build upon their socio-communicative development.

Previous literature on ASD has shown that behaviors such as coordinated eye contact, joint attention (Mundy and Crowson, 1997; Warreyn et al., 2005; Whalen et al., 2006) and dyadic orienting (Leekam and Ramsden, 2006; Koegel et al., 2009a) are important precursors for communication and socialization. In the current study, we were able to evoke social behaviors using sung directives which may serve as a simple albeit effective interventional medium to enhance social interaction and communication in children with ASD. Our findings show that singing based directives not only improved socio-communicative behaviors such as social gesture (“hi five”) and eye contact, but also improved non-social behaviors such as performance on a play activity. This suggests that song may not only be engaging, but also provide a communicative scaffold for children with ASD and help in the development of their social skills. This suggests that sung speech may play an important role for children with ASD by engaging them in interactive play activities and increasing attention, compliance, and socio-communicative skills. The findings from our study corroborate the results obtained in previous research that has used song as a tool for increasing social skills in children with autism (Stevens and Clark, 1969; Buday, 1995; Brownell, 2002; Pasiali, 2004; Kern and Aldridge, 2006; Finnigan and Starr, 2010).

Since ASD is conceptualized largely as a disorder of social impairment leading to delay in communication and other developmental milestones (Garfin and Lord, 1986; Koegel et al., 1992), most standard therapeutic interventions in ASD, aim at methods to enhance the development of these delayed skills. However, to learn any skill which is not driven by innate motivation, the child is required to engage with the therapist who leads the intervention. This in itself has been and remains an obstacle facing many therapeutic approaches.

Considering the rehabilitative potential of music therapies in facilitating neural plasticity as well as its intrinsic reward value, recent research in neuroscience has provided a robust biomedical perspective for clinical investigation of music therapies in various populations with psychiatric disorders. However, till date there are only few studies which have made an attempt to translate neuroimaging findings in a behavioral context and measure the efficacy of such interventions (e.g., Wan et al., 2010a, 2011; Wan and Schlaug, 2010). For instance, Wan et al. (2011) tested the efficacy of music making on expressive communication in non-verbal children with ASD, using a novel method called Auditory-Motor Mapping Training. This was motivated by previous neuroimaging studies that had suggested that the mirror neuron system responsible for imitative behaviors is implicated in ASD (Hadjikhani et al., 2006). In contrast to the Wan et al. (2011) study which focused on expressive communication and speech output in non-verbal children with ASD, our current investigation emphasized on the use of spoken and sung conditions in the receptive domain, particularly on socio-communicative responsiveness, contingent on engagement and motivation of the participants. This study was motivated by recent neuroimaging research which showed that neural pathways are preserved for sung word perception in children with ASD (Sharda et al., 2015) and was a direct follow-up from its findings. As suggested earlier, in another independent study, the neural networks for song processing remain intact and are more effectively engaged in the autistic brain than spoken words (Lai et al., 2012). The findings from our current behavioral study reaffirm such neurophysiological explanations for enhanced behavioral response to sung directives as compared to spoken instructions.

Future studies exploring the potential of song-based interventions could benefit from building upon the findings from this study. Despite the potential of our findings, there were some limitations of this study. Specifically, despite being a powerful design to conduct preliminary studies, a single-case design in which each participant acts as his own control (Barlow and Hayes, 1979; Barlow and Herson, 1984; Scruggs et al., 1987; Horner et al., 2005; Kennedy, 2005) cannot account for the generalization of results to other settings such as home, classroom or community. Therefore, it is not known whether these improvements would generalize and skills would transfer to other domains, since generalization to a new situation is of particular difficulty for children with autism (Jordan and Powell, 1995). Secondly, there was considerable variability in the data collected for each condition (Figure 1). Consequently, the treatment sung condition did not show any stable trend within the duration of the program, which might be due to the participants' volatility and other factors (Figure 2). Thirdly, since there was no clear order of ability between child A, B, and C, any trends that were observed were hard to interpret and depended on the measure (VABS vs. SRS) used (Figure 3). Therefore, it was difficult to make any generalized conclusions regarding the relationship between overall socio-communicative functioning and responsiveness to sung stimuli. Additionally, child B showed ceiling effects with very high responses particularly in the social gesture behavioral category (91% in spoken and near 100% in sung conditions), which was reflected in the low difference score of 0.04. A larger sample would help to clarify the situation in future research and lead to more statistically robust findings as indicated in previous music intervention studies (Geretsegger et al., 2014).

Future studies could replicate the current findings using larger samples to establish the validity of song as a therapeutic interventional medium to improve social responsiveness and communication. An individualized strategy which uses preferred melody, holds a promising role since preferred activities might be motivating and engaging context for children with autism (Koegel et al., 1987; Koegel and Koegel, 2006). In addition, future studies might also focus on determining which acoustic or musical elements of singing such as pitch, rhythmic pattern or tempo, are most salient in evoking a differential response from the children with ASD. However, the present study provides further empirical support to the anecdotal claims that the children with autism tend to be more engaged by music and songs than speech. Further explorations in this direction would lead to the development of song as a simple and effective interventional tool for children with ASD.

### Conflict of interest statement

The authors declare that the research was conducted in the absence of any commercial or financial relationships that could be construed as a potential conflict of interest.

## Supplementary material

Supplementary Figure S1 **Representative spectrograms for spoken vs. sung directives**. Spectrographic representations of spoken vs. sung directive (“Hi five!”) reflect the similarity of content in overall structure but differences in spectral distribution, in particular, the increased tonality of the sung as compared to the spoken directive.

Supplementary Audio Clips S1, S2 **Representative audio clips for verbal directive in spoken and sung conditions**. Audio clips of verbal directive “Hi five!” in spoken condition (S1) and sung condition (S2).
