# Supplementary figures and images for: The effect of sung speech on socio-communicative responsiveness in children with autism spectrum disorders
Source: Front Hum Neurosci. 2015 Oct 29;9:555. doi: 10.3389/fnhum.2015.00555 (PMC4624858; doi:10.3389/fnhum.2015.00555)

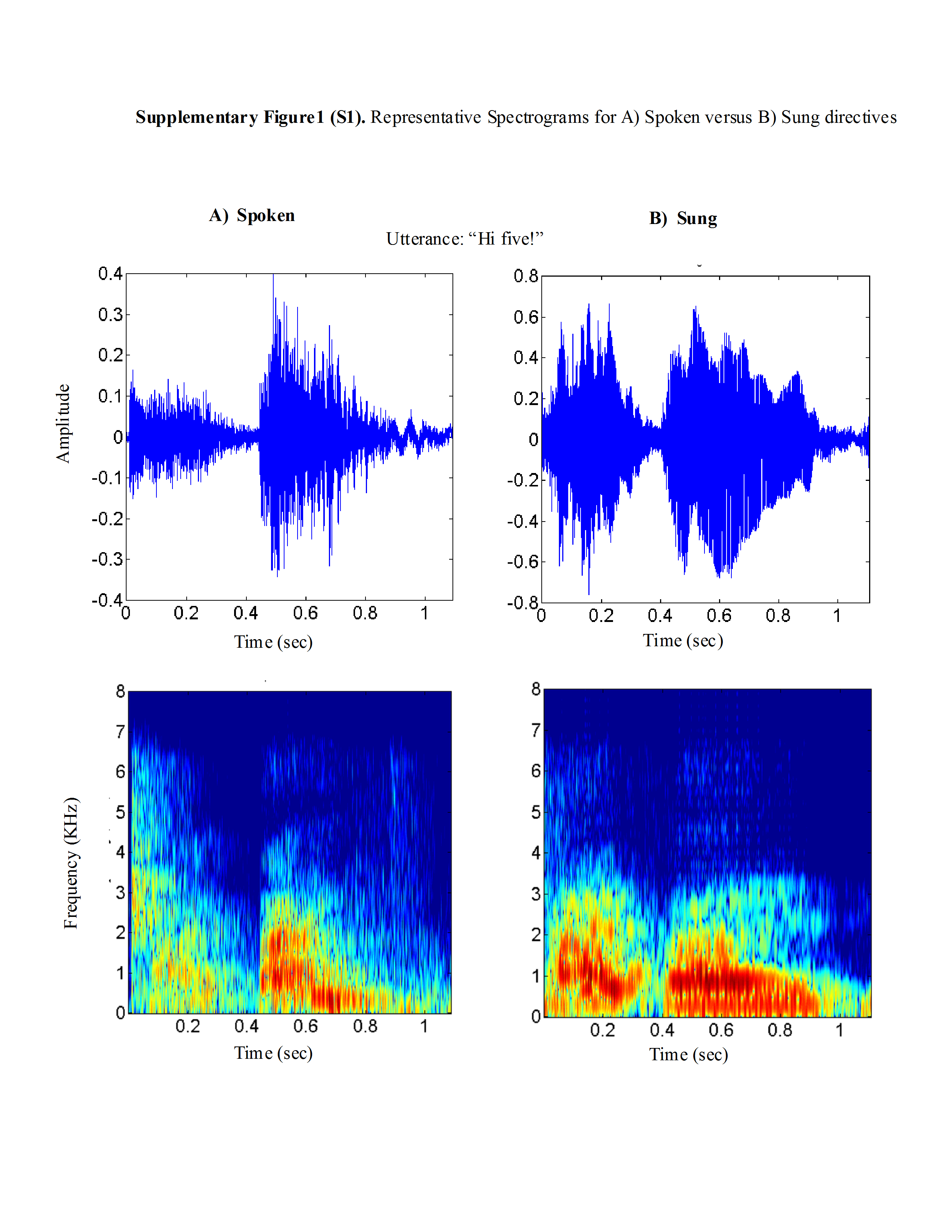

Supplement: Supplementary Figure S1 — Representative spectrograms for spoken vs. sung directives. Spectrographic representations of spoken vs. sung directive (“Hi five!”) reflect the similarity of content in overall structure but differences in spectral distribution, in particular, the increased tonality of the sung as compared to the spoken directive. [file Image1.TIF]
